# Need to focus on inhibitory activity of benzimidazole analogues against indolamine 2,3-dioxygenase-1 (IDO-1)

**DOI:** 10.17179/excli2022-4988

**Published:** 2022-06-28

**Authors:** Akshansh Sharma, Rajiv Tonk, Ravi Shekhar, Sushil Dohare, Deepak Kumar

**Affiliations:** 1 Department of Pharmaceutical Chemistry, School of Pharmaceutical Sciences, Shoolini University, Solan, Himachal Pradesh 173 229, India; 2 Department of Pharmaceutical Chemistry, School of Pharmaceutical Sciences, Delhi Pharmaceutical Sciences & Research University, New Delhi 110017, India; 3 Department of Pharmacy, Dr Bhimrao Ambedkar University, Agra, UP- India; 4 Department of Epidemiology, Faculty of Public Health & Tropical Medicine, Jazan University, Saudi Arabia

## ⁯

In this letter, substantial evidence for the potent antitumor activity of benzimidazole analogues as Indolamine 2,3-dioxygenase-1 (IDO1) inhibitors has been presented. Zhang et al. (2021[4]) recently published article piqued our interest in knowing whether benzimidazole derivatives are potent IDO1 inhibitors. The compound *6-fluoro-4-(4-((R)-1-(5-fluoro-1H-benzo[d]imidazol-2-yl)cyclohexyl)quinoline* has low metabolic stability but high cellular activity [HeLa/M109 IC_50 _= 0.0030.001/0.0220.021 M], which leads to the development of more stable and effective benzimidazole derivatives. Therefore, there is a significant demand to identify novel techniques for stimulating the anticancer immune system by enhancing the flexibility, productivity, and persistence of immunotherapy.

Serafini et al. (2021[3]) found IDO1 expression or activity in a variety of cancer cells, which is associated with a lower patient survival rate and a poor prognosis. To meet the challenge of finding new effective IDO1 inhibitors, medicinal chemists developed a structure-based virtual screening technique, that led to the discovery of promising benzimidazole analogues. IDO1 inhibitors have a binding mechanism that takes advantage of heme iron coordination and interlinkage with pocket A and B. Griglio et al. (2018[1]) unveiled a family of imidazothiazoles that exhibited a unique binding mechanism in the active site of IDO1 where the side chain obtrudes into an extra pocket C, a site of the formation of a significant hydrogen bond with Lys238 by ZINC 15, a database of widely available and drug-like substances.

With the help of OMEGA 2 software, maximum of 500 conformations were created for every single molecule, with a root mean square deviation (RMSD) of 0.8Å among conformers. The database was docked onto the IDO1 active site using HYBRID 30 (PDB structure 2D0T). This led to the discovery of *N-(3-((1H-Benzo[d]imidazol-1-yl) methyl) benzyl)-4-bromo-1H-pyrrole-2-carboxamide*, in which the benzimidazole moiety's nitrogen group coordinates with the heme group, the benzimidazole analogue is encapsulated in pocket A, and the side chain protrudes into pocket C. This tight packing allows for the development of a complex bonding network within the enzyme, resulting in the production of 3 hydrogen bonds and 1 halogen bond, with a high IC_50_ = 16 nM (inhibitory concentration) and K_d_ = 0.36 µM (strong binding interaction) in the a375 cell lines. When the bromopyrrole group of this compound is replaced by an indole moiety, *(N-(3-((1H-Benzo[d]imidazol-1-yl) methyl) benzyl)-1H-indole-2-carboxamide)* inhibits IDO1 in various cancer cell lines at low nanomolar levels and is more selective for IDO1. It may further lay the foundation for enhancing the efficiency of IDO1-targeted immunotherapy. Additionally, the PK profile is being optimized in their laboratory.

Another study by Hamilton et al. (2021[2]) showed potent action of the benzimidazole moiety in both mouse and human enzymes. Extensive or broad SAR showed promising compounds with outstanding PK and tumor targeted engagement. Few compounds demonstrated the highest potency, stable targeted engagement, adequate hERG margin, and better tumor penetration. These *were 4-Chloro-N-((R)-1-((1R,3S,5S,6r)-3-(5,6-difluoro-1H-benzo[d]-imidazole-1-yl)bicyclo[3.1.0]hexan-6-yl)propyl)benzamide *[IACS-9779]*, N-(4-Chlorophenyl)-2-((1R,3s,5S,6r)-3-(5,6-difluoro-1H-benzo[d]-imidazol-1-yl)bicyclo[3.1.0]hexan-6-yl) propanamide *[IACS70099]*, *and* N-(4-Chlorophenyl)-2-((1R,3s,5S,6r)-3-((6-fluoroquinolin-4-yl)-oxy) bicyclo [3.1.0]hexan-6-yl)propenamide *[IACS-70465]*. *The IACS-70099 study was discontinued because of toxicity, while the IACS-70465 profile was not completed due to the cancellation of the program. During a preliminary rat toxicology investigation, IACS-9779 demonstrated a higher safety profile than IACS-70099, indicating that it warrants further investigation.

Zhang et al. (2021[4]) introduced a chloro substituent to the phenyl ring of benzimidazole, and a nitrogen atom, which resulted in imidazopyridine and had a substantial increase in stability, preserving hWB activity [hWB IC_50_ = 0.039 μM]. The synthesized imidazopyridine, [HeLa/M109 IC_50_ = 0.002±0.002/0.003±0.002 μM] comparison to linrodostat showed more pronounced CYP (Cytochrome P450) inhibition and PXR (Pregnane X Receptor) activation across many isoforms; as a result, this was not further studied; hence IDO1 inhibitors could be better explored.

Even though the literature suggests benzimidazole is a preferred scaffold, its immense potential is underexplored. The lack of data on effective benzimidazole-based IDO1 inhibitors requires more research. This could remind researchers, editors, and peer reviewers of how important it is to look at these compounds as IDO1 inhibitors.

## Conflict of interest

The authors declare no conflict of interest.
